# To Explore the Mechanism and Equivalent Molecular Group of Radix Astragali and Semen Lepidii in Treating Heart Failure Based on Network Pharmacology

**DOI:** 10.1155/2021/5518192

**Published:** 2021-07-03

**Authors:** Yi-ding Yu, Yi-ping Xiu, Yang-fan Li, Juan Zhang, Yi-tao Xue, Yan Li

**Affiliations:** ^1^Shandong University of Traditional Chinese Medicine, Jinan 250014, China; ^2^Affiliated Hospital of Shandong University of Traditional Chinese Medicine, Jinan 250014, China

## Abstract

Radix Astragali and Semen Lepidii (HQ-TLZ) is a commonly used herbal medicine combination for treatment of heart failure, which has a good clinical effect. However, its active components and mechanism of action are not clear, which limits its clinical application and development. In this study, we explored the mechanism of action of HQ-TLZ in the treatment of heart failure based on network pharmacology. We obtained 11 active ingredients and 109 targets from the TCMSP database and SwissTargetPrediction database. Next, we constructed the action network and carried out enrichment analysis. The results showed that HQ-TLZ treatment of heart failure is primarily achieved by regulating the insulin resistance, erbB signaling pathway, PI3K-Akt signaling pathway, and VEGF signaling pathway. After inverse targeting, molecular docking, and literature search, we determined that the equivalent molecular groups of HQ-TLZ in the treatment of heart failure were quercetin and kaempferol. Based on network pharmacology, we reveal the mechanism of action of HQ-TLZ in the treatment of heart failure to a certain extent. At the same time, we determined the composition of the equivalent molecular group. This provides a bridge for the consistency evaluation of natural herbs and molecular compounds, which is beneficial to the development of novel drugs and further research.

## 1. Introduction

Heart failure (HF), the ultimate cause of most cardiovascular diseases, is the result of the filling or ejection of damaged ventricles caused by various cardiac structural or functional disorders [1]. Cardiac remodeling runs through the occurrence and development of heart failure and is one of the main factors determining cardiac function and prognosis. Previously, renin-angiotensin system inhibitor (RASI) was the first choice for the treatment of heart failure and improved cardiac remodeling, but the morbidity and mortality of patients remained high [2]. In order to find a safe and effective treatment, we turn to traditional Chinese medicine. HQ-TLZ is a commonly used and efficacious herbal combination for the treatment of heart failure [3], but its active components and mechanism of action are still unclear.

In 2008, a British pharmacologist Hopkins first proposed the concept of network pharmacology. Network pharmacology is a combination of bioinformatics, multimodal pharmacology, network data analysis, and computer technology to explore the association between drugs and diseases through the analysis of the “drug-targets-disease” interaction network [4]. Therefore, this study aims to analyze the mechanism of action of HQ-TLZ in the treatment of heart failure through the network pharmacology method and find the equivalent molecular groups of these two traditional Chinese herbals.

Equivalent molecular cluster is a new concept put forward by our research group in order to find a means of modernization of traditional Chinese medicine [5]. An equivalent molecular group is defined as a collection of compounds whose efficacy is equivalent to that of oral Chinese herbal decoction in the real world. Equivalent molecular groups can provide some ideas for drug mechanism research and new drug development.

## 2. Method

### 2.1. HQ-TLZ Active Ingredients Screening and Target Prediction

The Chinese medicine systematic pharmacology database (TCMSP) (http://tcmspw.com/tcmsp.php) is an open and comprehensive database of Chinese medicine ingredients and action targets. In clinical treatment, traditional Chinese herbals enter the body by oral administration. Oral bioavailability (OB) and drug-likeness (DL), two ADME-related models, are the main variables affecting the absorption of drugs from the gastrointestinal tract. Therefore, we screened bioactive components under the conditions of OB ≥30% and DL ≥ 0.18. PubChem (https://pubchem.ncbi.nlm.nih.gov/) is a chemical database module intended to promote small molecular data resources for public use. We use it to get compound SMILE expression. Then, we used the SwissTargetPrediction database (http://www.swisstargetprediction.ch/) to predict the target of the active ingredient, and we selected the probability greater than 0.5 as the target of the active ingredient of HQ-TLZ.

### 2.2. Heart Failure Target Screening

We take “heart failure” as keywords in Genecards database search (https://www.genecards.org/) to retrieve the heart failure targets.

### 2.3. Direct-Action Target Screening

We intersected action targets of HQ-TLZ active ingredients and heart failure disease action targets to obtain the direct-action targets of HQ-TLZ active ingredients for the treatment of heart failure.

### 2.4. HQ-TLZ “Component-Target” Network Diagram Construction

We constructed the “compound-target” network diagram of HQ-TLZ by Cytoscape3.6.1. In the network diagram, “node” refers to the compound or target and “edge” refers to the relationship between the compound and the target.

### 2.5. Enrichment Analysis of Direct Targets

We input the targets into the David 6.8 database to obtain KEGG signaling pathway data and GO biological function data and analyze the results with *P* < 0.05.

### 2.6. Inverse Target Compounds

Based on the KEGG signaling pathway data, we analyzed the heart-failure-related pathways. We screened key compounds and targets on pathways based on the “compound-target” network diagram.

### 2.7. Molecular Docking

Structure-based docking studies were carried out by using Autodock Vina. 3D conformations of the 2 molecules were generated and minimized using the molecular mechanics (MM2) method. A cube of 20 Å around the binding site in the kinase domain was defined as the docking site. The number of modes was set to 20, and the exhaustiveness was set to 8. Other parameters were set to default. At last, Vina was utilized to connect the active ingredients with the target protein in turn, and Affinity was extracted. PyMol was utilized to analyze and plot the results. AutoDock Vina uses semiflexible molecular docking; that is, the pharmacophore is flexible while the protein remains rigid during the docking. The docking results are evaluated by a semiempirical free-energy function.

## 3. Result

### 3.1. Data Collections

After TCMSP retrieval, ADME parameter screening, and SwissTargetPrediction prediction target, 9 active components of HQ were obtained and 102 active targets were identified. Similarly, we obtained 6 active components and 102 target sites of TLZ. After summing up the obtained compounds and action targets and removing the duplicated values, 11 active components and 109 action targets of HQ-TLZ were finally obtained.

We retrieved 13,194 heart failure targets (both protein and RNA targets) from the Genecards database. We intersected the results of the two and obtained 102 direct-action targets.

### 3.2. Network Buildings

**Figure 1:**
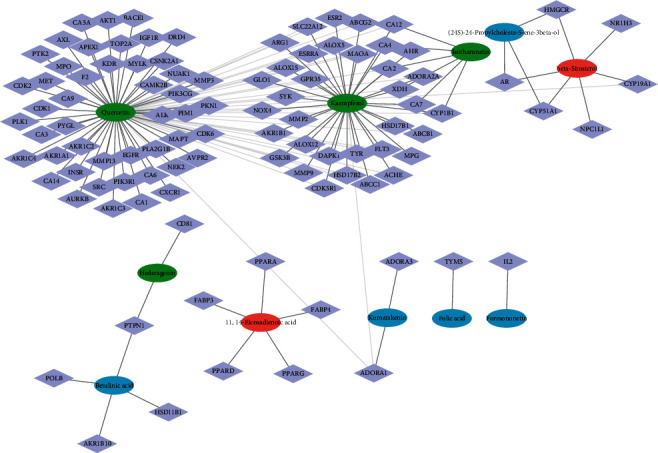
HQ-TLZ “component-target” network diagram. Among them, the circle represents the compound and the diamond represents the target. The blue circle is a compound unique to HQ, the red circle is a compound unique to TLZ, and the green circle is a compound common to both drugs.

We built the network diagram using Cytoscape's Clustermaker capabilities, as shown in Figure 1. The network diagram reflects the multicomponent and multitarget action characteristics of HQ-TLZ in the treatment of heart failure. The network diagram is mainly divided into two large modules and four small modules. Quercetin and kaempferol are located in the center of two large modules, suggesting that these two compounds may play a major role in the biological activity of HQ-TLZ. The third module suggested that there might be a synergistic effect between HQ-TLZ, but whether this biological process is related to heart failure still needs to be further analyzed. The fifth and sixth modules indicated that HQ-TLZ had their own targets, which may be one of the reasons for the difference of sexual taste meridian between HQ and TLZ in traditional medical theory.

### 3.3. Enrichment Analysis of Data

**Figure 2:**
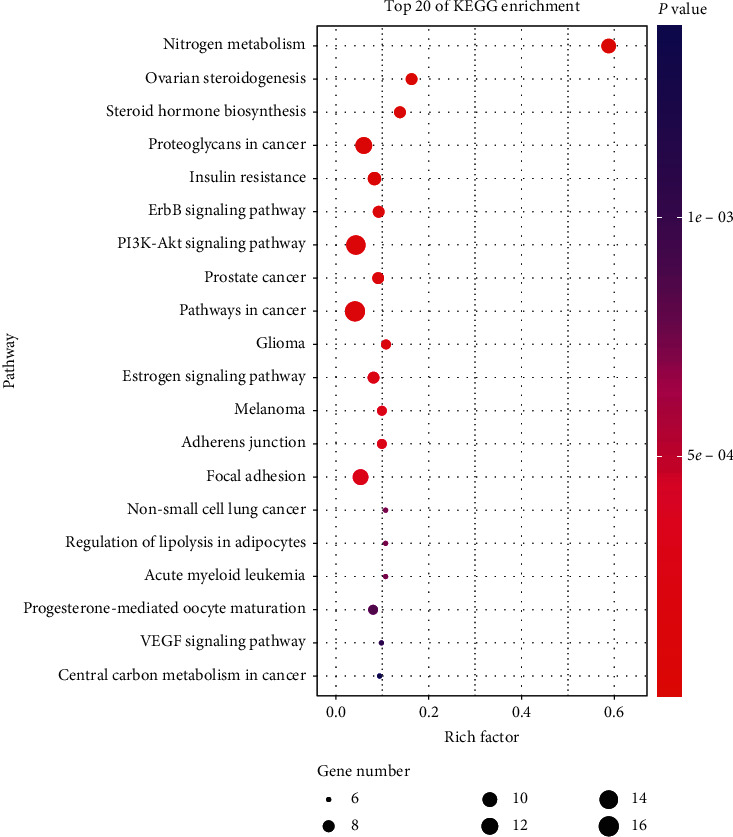
The top 20 of enrichment results.

KEGG analysis showed that the key targets of HQ-TLZ in the treatment of heart failure were enriched in 68 pathways, and 50 of them had *P* value less than 0.05. Among these pathways, the insulin resistance, erbB signaling pathway, PI3K-Akt signaling pathway, and VEGF signaling pathway are closely related to this study. We show the top 20 pathways for enrichment results, detailed in Figure 2.

**Figure 3:**
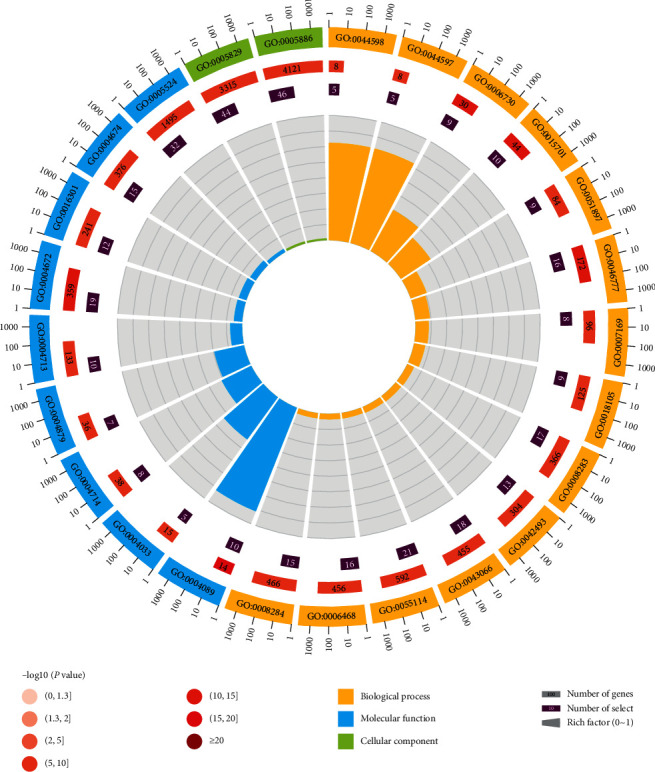
The top 25 of GO notes.

GO functional enrichment analysis showed that there were 247 GO annotations with *P* < 0.05. We screened out the top 25 of GO notes based on *P* value, count, and pop hits, as shown in Figure 3. In the figure, 14 GO notes are related to Biological Process (BP), 9 are related to Molecular Function (MF), and 2 are related to Cellular Component (CC). The main functions involved are mostly related to various protein activities, the doxorubicin metabolic process, the daunorubicin metabolic process, the one-carbon metabolic process, etc.

### 3.4. Inverse Target Compounds

After analyzing KEGG signaling pathways, we got the 24 targets associated with heart failure. Among them, we obtained 6 key targets through the screening of protein location and degree, named PIK3CG, AKT1, PIK3R1, GSK3B, CAMK2B, and IGF1R.

After network screening, we obtained two key compounds, quercetin and kaempferol.

### 3.5. Molecular Docking Results

**Figure 4:**
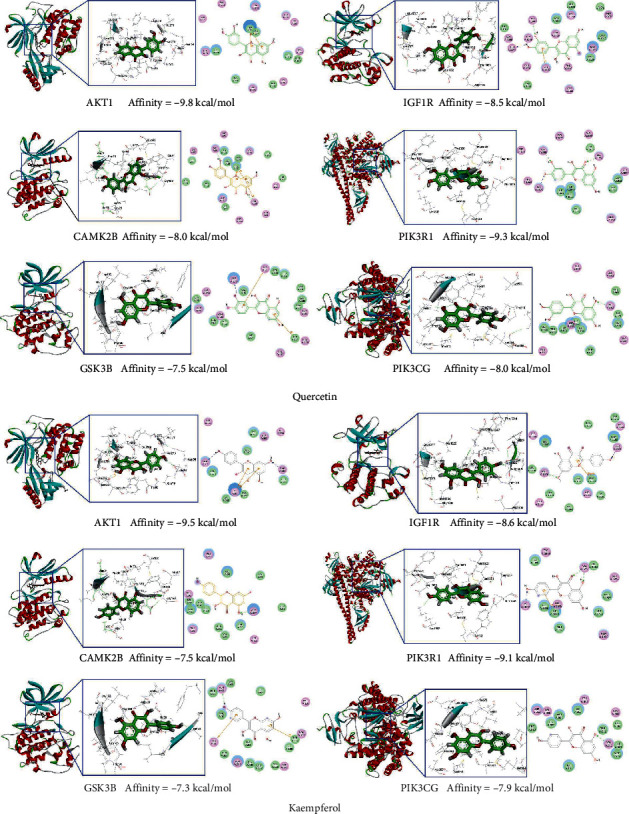
Docking results. It is generally believed that an affinity less than −5 kcal/mol indicates a better binding.

AutoDock Vina evaluates the binding ability of small molecules to proteins mainly by affinity. Affinity less than 0 indicates that the ligand can spontaneously bind to the receptor, and the smaller the value is, the higher the affinity is and the easier it is for the active component to bind to the receptor. The docking results and affinity are shown in Figure 4.

## 4. Discussion

Treatment for heart failure has evolved in three stages. From the anatomical stage of cardiac strengthening and diuresis, to the hemodynamic stage of cardiac strengthening, diuresis, and vasodilation, and finally, to the present neuroendocrine stage, people have gradually realized that the essence of heart failure is ventricular remodeling. Overcompensation and persistence of neuroendocrine will be an adverse prognostic factor. Therefore, reversing ventricular remodeling and regulating neuroendocrine system are the cornerstone of the treatment of heart failure [6]. Owing to the characteristics of multicomponent and multitarget, TCM has certain advantages in this respect.

We know that systemic insulin resistance is a risk factor for heart failure and is independent of coronary artery disease [7]. Studies have shown that patients with heart failure have similar fasting blood-glucose levels as normal people, but higher plasma insulin levels [8]. Recent studies have found that myocardial insulin resistance is also an important factor in the occurrence and development of heart failure. Moreover, due to the lack of understanding of myocardial insulin resistance, the prognosis of patients may not be improved by the administration of certain antiglucose drugs [9].

The PI3K-Akt signaling pathway is one of the main signaling cascade pathways downstream of IGF1R. On binding to its ligand, insulin and insulin-like growth factor-1 (IGF-1) receptors undergo autophosphorylation, which increases their tyrosine kinase activities. Tyrosine phosphorylation and activation of the docking proteins insulin receptor substrates 1 and 2 (IRS1/2) engages regulatory subunits of the phosphatidylinositol-3-kinase (PI3K) to regulate the PI3K-Akt signaling pathway [10].

Inhibiting the PI3K-Akt signaling pathway has been shown to improve cardiac hemodynamic impairment and fibrosis [11]. Meanwhile, GSK3B, a direct substrate of the PI3K-Akt signaling pathway, will be activated by a number of ways, including phosphorylation of GATA4, which regulates cardiac hypertrophy, phosphorylation of eIF2B to inhibit protein synthesis, and phosphorylation of glycogen synthase to inhibit glycogen synthesis to prevent cardiac hypertrophy and inhibit the progression of ventricular remodeling [12–14].

The ErbB signaling pathway is regulated by the widely expressed signaling molecule neuroregulatory protein-1 (NRG-1). NRG-1/ErbB signaling is important for maintenance of cardiac function in adult organisms. Various mechanisms are believed to be involved in this process, including promotion of cardiac myocyte survival, improvement of sarcomeric structure and cell-cell adhesion, and maintenance of Ca2+ homeostasis [15]. Meanwhile, continued activation of the ErbB signaling pathway silences downstream Akt [16], thereby inhibiting the progression of ventricular remodeling. However, studies have shown that ERBB2 suppression is a common anticancer strategy, with 25% of breast cancer patients with ERBB2 over expression [17]. Therefore, more evidence is required to determine whether activation of the ErbB signaling pathway increases the risk of cancer in patients with heart failure.

Vascular endothelial growth factor (VEGF) plays an important role in mediating normal cardiac function by maintaining vascular homeostasis. VEGF maintains vascular homeostasis mainly through the following mechanisms: improve the sensitivity of blood vessels to nerve response, improve the permeability of blood vessels, promote the generation and stability of new blood vessels, recruit stem cells, and promote their homing [18]. The research of Meng-ying He showed that when the VEGF signaling pathway was inhibited, it would lead to the imbalance of vascular homeostasis, which would lead to the generation of heart failure [19].

Traditional medicine (TM) is being used more frequently all over the world. However, most often, these are choices made by the patient. These medical traditions have a unique understanding of physiology, pathogenesis, pharmacology, and pharmaceuticals, which are different from Western biomedicine [20]. These differences lead to communication difficulties between traditional medicine and modern medicine. At the same time, it is difficult for traditional medicine to enjoy the achievements of modern science and technology. Also, the characteristic of multicomponent acting on multitarget poses a dilemma for the evaluation of therapeutic efficacy of herbal medicines [21]. For this reason, we put forward the concept of the equivalent molecular group. Our research group believes that no matter what role Chinese herbal medicine plays in TCM theory, from the perspective of modern science, the compounds in Chinese herbal medicine must have some kind of reaction in vivo, just like Tu Youyou's research. In Chinese medical terms, *Artemisia annua* offers the functions of cooling and detoxifying blood, eliminating osteopyrexia and fever, freeing from summer heat, preventing recurrence of malaria fevers, and removing jaundice. But, from the perspective of modern science, Tu Youyou discovered that artemisinin in *Artemisia annua* can kill malaria parasites [22]. We can think that artemisinin is the equivalent molecular group of *Artemisia annua* for treating malaria. Even so, research on other traditional Chinese medicines is not as clear as that on *Artemisia annua*. Therefore, we decided to first look for the equivalent molecular group of Chinese medicine to treat a single disease. After component screening, target prediction, enrichment analysis, and inverse targeting, we concluded that the equivalent molecular groups of HQ-TLZ in the treatment of heart failure were quercetin and kaempferol.

Molecular docking results showed that quercetin and kaempferol could spontaneously bind to key targets in the pathway. When kaempferol and quercetin bind to these proteins, the proteins lose the chance to bind to other compounds and are expressed in an inhibited state. Considering the location of key targets in these pathways, we hypothesized that quercetin and kaempferol could inhibit these four pathways to some extent. To understand the relationship between quercetin and kaempferol and these pathways, we searched the relevant literature. The retrieval results were in line with our prediction.

Quercetin significantly attenuated EGF and TGF-a-induced growth and phosphorylation of ErbB2, ErbB3, cRaf, MAPK kinase 1/2 (MEK1/2), MAPK, Elk1, and Akt1 [23]. At the same time, quercetin induces cell death via downregulation of VEGF signaling pathways and mitochondria-mediated apoptosis in cells [24, 25]. Meanwhile, quercetin can also inhibit the PI3K-Akt signaling pathway and reduce insulin resistance [26–28].

Kaempferol can inhibit the VEGF signaling pathway to play an antiangiogenesis role and, thus, play a certain role as an antitumor agent and in reducing diabetic retinopathy [29, 30]. Similarly, kaempferol can inhibit the PI3K-Akt signaling pathway and increase insulin sensitivity and reduce insulin resistance [31–34].

From the abovementioned results, the effects of quercetin and kaempferol on the ErbB signaling pathway and VEGF signaling pathway are not conducive to the treatment of heart failure. However, its role in the insulin resistance and PI3K-Akt signaling pathway is positive. It seems difficult to show what the combined effects of quercetin and kaempferol are. Then, we looked at the relationship between quercetin and kaempferol with heart failure.

Our literature search results indicate that there are no clear studies about quercetin and kaempferol with heart failure, but there are experiments showing that both quercetin and kaempferol can inhibit angiotensin-II-induced ventricular remodeling [35, 36]. This suggests that quercetin and kaempferol may benefit patients with heart failure.

We know that heart failure has the same risk factors as cancer. Also, patients with heart failure have a higher rate of cancer than healthy control populations [37]. It may be that quercetin and kaempferol can reduce the risk of cancer in patients with heart failure, but more experiments and studies are needed to confirm this view.

There are still some shortcomings in this study. Due to the limitations of network pharmacology, it is difficult to analyze the dosage of compounds. More preparation is needed in the next step of consistency research.

## 5. Conclusions

In conclusion, this study has found the potential mechanism of HQ-TLZ in the treatment of heart failure based on the network pharmacology method, which is consistent with the mechanism of action of multicomponent and multitarget of traditional Chinese medicine. At the same time, it is reasonable to assume that the equivalent molecular group of HQ-TLZ is the collection of quercetin and kaempferol. This provides a bridge for the consistency evaluation of natural herbs and molecular compounds, which is beneficial to the development of novel drugs and further research.

## Data Availability

The data used to support the findings of this study are available from the corresponding author upon request.
